# A case of sigmoidal mesenteric lipoma with preoperative diagnosis of ovarian tumor: A case report

**DOI:** 10.1016/j.radcr.2021.05.022

**Published:** 2021-06-13

**Authors:** Shohei Tanabe

**Affiliations:** Department of Obstetrics and Gynecology, Maternal and Perinatal Care Center, Seirei Hamamatsu General Hospital, 2-12-12 Sumiyoshi, Naka-ku, Hamamatsu City, Shizuoka, 430-8558, Japan

**Keywords:** Sigmoidal mesenteric lipoma, Ovarian tumor

## Abstract

Mesenteric lipomas are rare tumors, with fewer than 50 reported cases. A 42-year-old woman with no significant medical history was referred to our hospital for surgical removal of submucosal myoma and a left ovarian tumor that was diagnosed at a different clinic. Preoperative magnetic resonance imaging findings suggested a differential diagnosis of fibroma, Brenner's tumors, and mature teratomas; however, the intraoperative findings showed a sigmoidal mesenteric tumor adherent to the left ovary. Pathological examination revealed degenerated adipose tissue, and the patient was diagnosed with sigmoidal mesenteric lipoma. When a degenerated sigmoidal mesenteric lipoma is adherent to the ovary, it must be differentiated from an ovarian tumor.

## Introduction

Mesenteric lipomas are rare tumors, with fewer than 50 reported cases. Because these tumors are soft, they become symptomatic only after enlarging and pressing on surrounding tissues [1]. Consequently, mesenteric lipomas are frequently found during surgery. We recently encountered a case who was preoperatively diagnosed with an ovarian tumor, but the intraoperative and pathological findings led to the diagnosis of sigmoidal mesenteric lipoma. To the best of our knowledge, this is the first case report that requires differentiation between sigmoidal mesenteric lipoma and ovarian tumor; therefore, we report it with some discussion.

## Case report

**Fig. 1:**
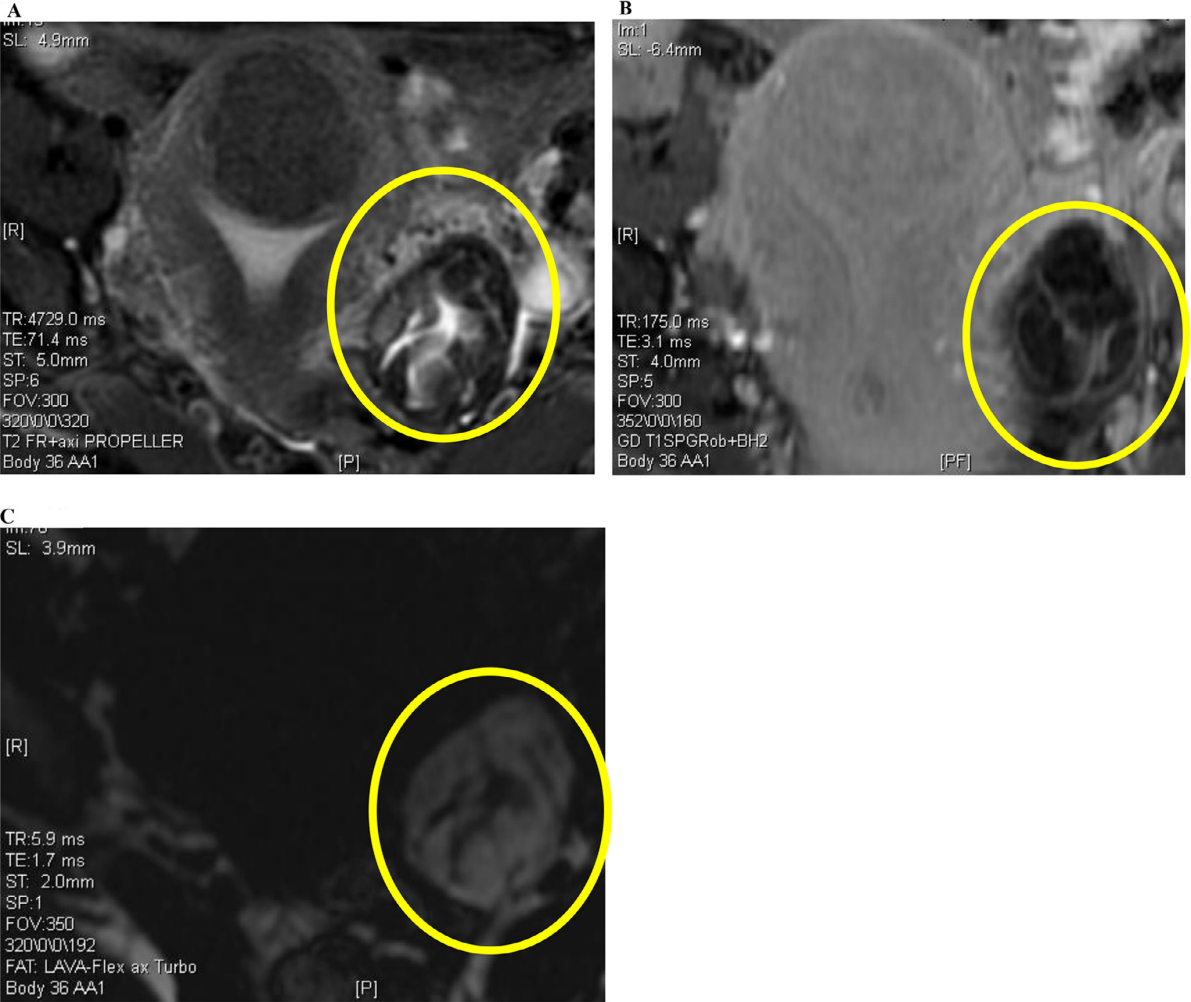
T2-weighted (A), T1-weighted (B), and fat-suppressed T1-weighted (C) images. Circled sigmoid mesenteric lipoma resembling an ovarian tumor.

A 42-year-old woman with no significant medical history visited her previous doctor complaining of heavy periods and was diagnosed with submucosal fibroids by transvaginal ultrasonography. Magnetic resonance imaging (MRI) at our hospital (Figs. 1A, B, and C) revealed a low signal for the left ovarian tumor on T2-weighted images, consistent with a fibroma or Brenner's tumor. However, a high signal on T1-weighted images and a low signal on fat-suppressed T1-weighted images were suggestive of a main, fatty component. We therefore considered a mature teratoma during differential diagnosis. Mesenteric lipomas are rare and consequently were excluded from the differential diagnosis.

**Fig. 2:**
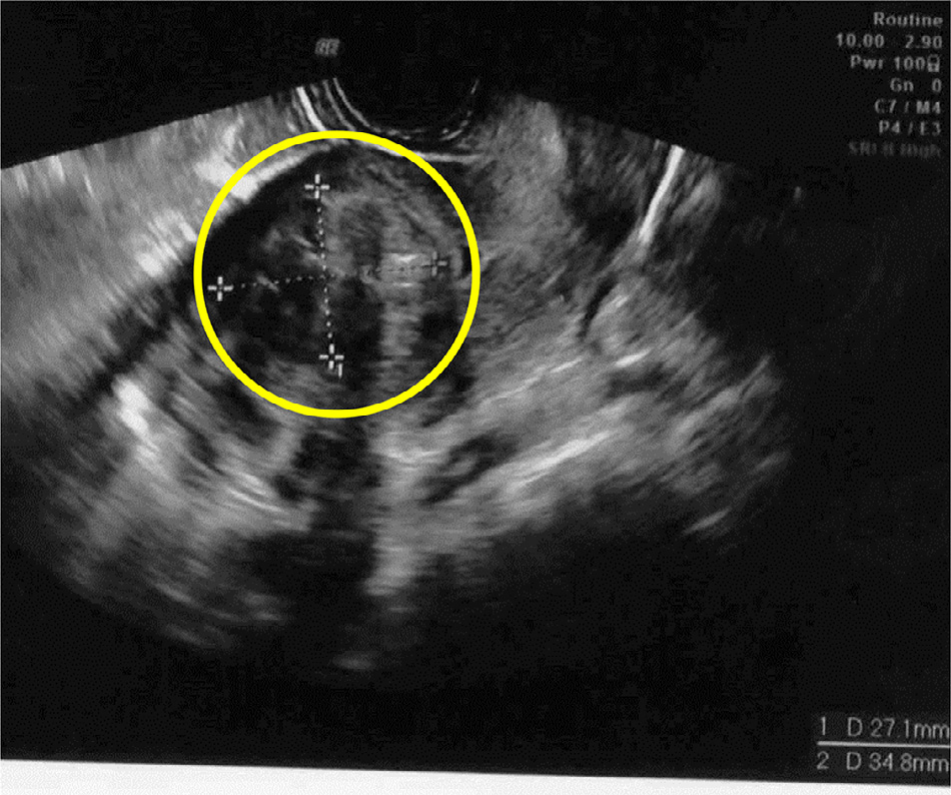
A substantial tumor (circled), 27.1 mm × 34.8 mm in size.

The patient was referred to our hospital for surgery. Transvaginal ultrasound showed a 27.1 mm × 34.8 mm tumor (Fig. 2). After preoperative administration of two doses of gonadotropin-releasing hormone agonist, total laparoscopic hysterectomy, left ovarian tumor resection, and right oophorectomy were planned. Four months passed between initial diagnosis and surgery at our hospital.

**Fig. 3:**
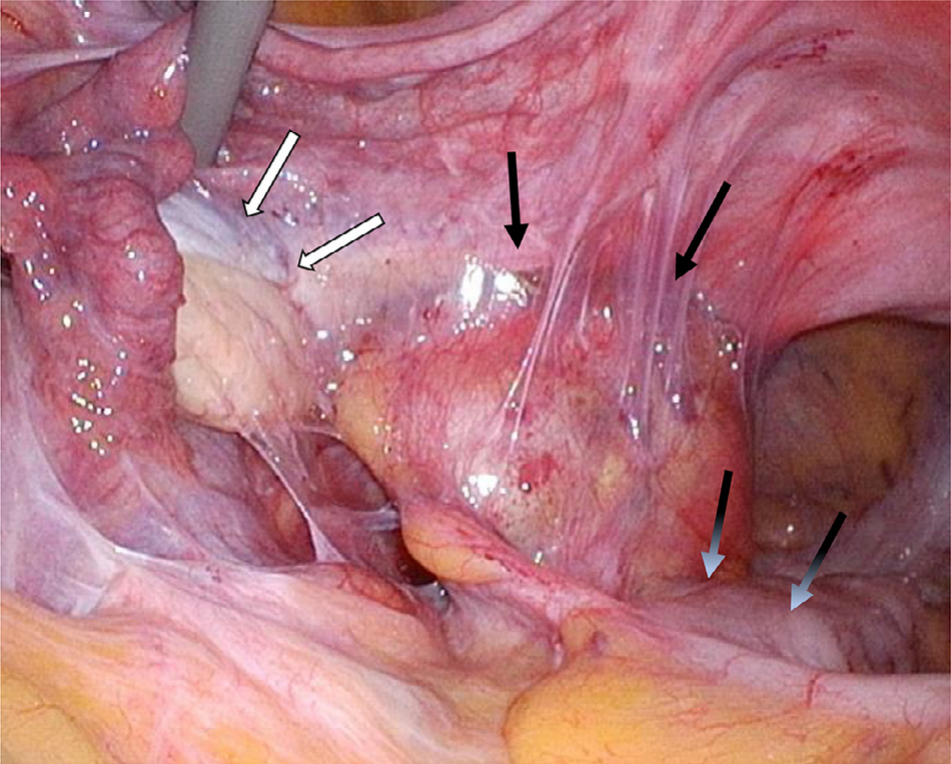
White arrow: left ovary; black arrow: sigmoid colon mesenteric tumor; black and white arrow: sigmoid colon

**Fig. 4:**
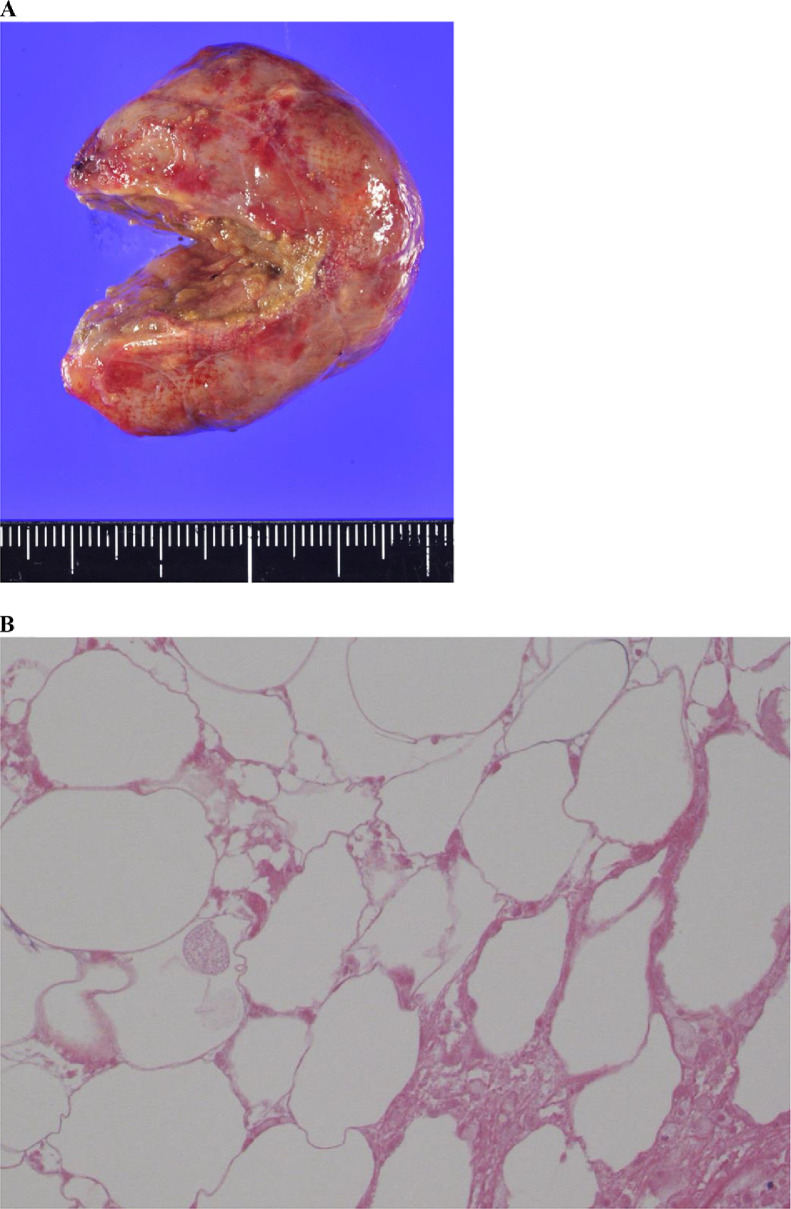
A. Macroscopic pathological finding: Tumor with the degenerated fatty component; B. Staining: hematoxylin and eosin. Original magnification: 600 × . The image of the fatty component reveals fatty tissue with degeneration, necrosis, fibrosis, and macrophage reaction.

The intraoperative images are shown in Fig. 3. Although a left ovarian tumor was diagnosed preoperatively, the intraoperative findings showed that the tumor-derived from the sigmoid colon mesentery adhered to the uterus's posterior wall and the left ovary. The tumor was removed by detaching it from the surrounding connective tissue. The tumor was covered with a white membrane and contained degenerated adipose tissue. The surgical plan was modified to include total laparoscopic hysterectomy, bilateral oophorectomy, and mesenteric tumor resection. The operation time was 129 minutes with 20 mL blood loss.The 4-cm tumor weighed 20 gm. The pathological findings are shown in Fig. 4A and B. These findings include fatty tissue with degeneration and necrosis with fibrosis and macrophage reaction, consistent with a lipoma.

Since the sigmoidal mesenteric tumor was composed of adipose tissue, we diagnosed the patient with a sigmoidal mesenteric lipoma. She was discharged from the hospital without any postoperative complications and is currently being treated as an outpatient at our hospital.

## Discussion

The mesenteric tumor is a very rare primary tumor with an estimated incidence of 1/100,000. Because the mesentery contains connective, vascular, adipose, lymphoid, and neural tissues, tumors can originate from any one of these tissues [2]. Mesenteric lipomas are generally soft and slow-growing and can be difficult to diagnose because symptoms typically only emerge after the tumor grows [3]. Consequently, mesenteric lipomas are rarely symptomatic and are often diagnosed incidentally during laparoscopy or laparotomy [4]. Lipomas show characteristic findings on imaging. They have no contrast effect; therefore, contrast-enhanced abdominal CTscans produce CT values equivalent to those of the surrounding fat without a contrast effect [5]. CT was not performed in the present case, and MRI showed a low signal on fat-suppressed images. In this case, the low signal on the fat-suppressed image indicated the presence of a fatty component. We initially considered an ovarian tumor and a mature teratoma was initially listed as the differential disease. Because the T2-weighted images mainly showed low-signal areas, fibroma and Brenner's tumor were also considered. Intraoperatively, we found a mesenteric lipoma adherent to the normal left ovary, which appeared as an ovarian tumor on MRI. In addition, the pathological findings showed that the fatty tissue had degenerated. Even though the tumor was a lipoma, the low signal on T2-weighted imaging resulted from adipose tissue degeneration.

Mesenteric and ovarian tumors may require differentiation based on their proximity to the ovary. Furthermore, mature teratoma(a type of ovarian tumor)contains fatty components. Previous investigations found it difficult to differentiate between a mature teratoma and a mesenteric lipoma of the ileum based on imaging findings alone [6]. Our case's mesenteric lipoma adhered to the normal ovary, and the imaging findings led to the diagnosis of ovarian tumor. We included a mature teratoma in the differential diagnosis because of the tumor's fatty component, but the presence of only a fatty component and no calcification or a hairball was atypical for mature teratoma.

Patients are managed with laparoscopic resection for small mesenteric lipomas and laparotomy for larger tumors [7]. Laparoscopic surgery is superior for postoperative pain control and recovery speed; however, laparotomy is recommended for more difficult cases for safety reasons [8]. However, laparoscopic surgery is considered sufficient in cases, such as the present case, of incidental detection.

In conclusion, if a degenerated sigmoid mesenteric lipoma is adherent to the ovary, other ovarian tumors such as fibroma, Brenner tumor, or mature teratoma should be considered.

## References

[1] Buono GD, Ricupati F, Amato G (2020). Small bowel volvulus due to a large intestinal lipoma: A rare case report. Int J Surg Case Rep.

[2] Abdallah FK, Lakkis RS, Terro JJ (2020). Giant adult mesenteric lipoma: a rare cause of chronic abdominal distention and discomfort. Case Rep Surg.

[3] Dufay C, Abdelli A, Le Pennec V (2012). Mesenteric tumors: Diagnosis and treatment. J Visc Surg.

[4] Cha JM, Lee JI, Joo KR (2009). Giant mesenteric lipoma as an unusual cause of abdominal pain: a case report and a review of the literature. J Korean Med Sci.

[5] Suga Y, Abdi E, Bekele M (2019). Giant mesenteric lipoma causing small bowel volvulus: a case report. Ethiop J Health Sci.

[6] Srinivasan KG, Gaikwad A, Ritesh K (2009). Giant omental and mesenteric lipoma in an infant. African J Paediatr Surg.

[7] Sherer DM, Lysikiewicz A, Chambers JT (2002). Large mesenteric lipoma ultrasonographically mimicking a mature cystic teratoma during pregnancy. J Ultrasound Med.

[8] Watt DG, Sanjay P, Walsh SV (2012). Mesenteric lipoma causing small bowel perforation: a case report and review of literature. Scott Med J.

